# Plasma metabolomics identifies differing endotypes of recurrent wheezing in preschool children differentiated by symptoms and social disadvantage

**DOI:** 10.1038/s41598-024-66878-1

**Published:** 2024-07-09

**Authors:** Anne M. Fitzpatrick, Jocelyn R. Grunwell, Hina Gaur, Seibi Kobara, Rishikesan Kamaleswaran

**Affiliations:** 1 Department of Pediatrics, Emory University School of Medicine, 2015 Uppergate Drive Office #340, 30322 Atlanta, Georgia; 2 Division of Pulmonary Medicine, Children’s Healthcare of Atlanta, Atlanta, Georgia; 3 Division of Critical Care Medicine, Children’s Healthcare of Atlanta, Atlanta, Georgia; 4 Department of Biomedical Informatics, Emory University School of Medicine, Atlanta, Georgia; 5 Department of Surgery, Duke University School of Medicine, Durham, NC USA

**Keywords:** Asthma, Wheezing, Children, Metabolomics, Phenotype, Endotype, Diagnostic markers, Paediatric research, Translational research

## Abstract

Preschool children with recurrent wheezing are a heterogeneous population with many underlying biological pathways that contribute to clinical presentations. Although the morbidity of recurrent wheezing in preschool children is significant, biological studies in this population remain quite limited. To address this gap, this study performed untargeted plasma metabolomic analyses in 68 preschool children with recurrent wheezing to identify metabolomic endotypes of wheezing. K-means cluster analysis was performed on metabolomic dataset including a total of 1382 named and unnamed metabolites. We identified three metabolomic clusters which differed in symptom severity, exacerbation occurrence, and variables associated with social disadvantage. Metabolites that distinguished the clusters included those involved in fatty acid metabolism, fatty acids (long chain monounsaturated fatty acids, long chain polyunsaturated fatty acids, and long chain saturated fatty acids), lysophospholipids, phosphatidylcholines, and phosphatidylethanolamines. Pathway analyses identified pathways of interest in each cluster, including steroid metabolism, histidine metabolism, sphingomyelins, and sphingosines, among others. This study highlights the biologic complexity of recurrent wheezing in preschool children and offers novel metabolites and pathways that may be amenable to future study and intervention.

## Introduction

Recurrent wheezing is a common and troubling symptom in preschool children. Nearly 50% of all preschool children have one episode of wheezing^1,2^ and 20% of these children have recurrent wheezing before 6 years of age^2^. Recurrent wheezing in preschool children is highly problematic^3^ and results in disproportionate morbidity, with twice the rate of emergency department visits and three times the rate of hospitalization compared to older children with asthma^4^. As a result, recurrent wheezing in preschoolers is associated with profound costs (> $6 billion annually)^5–7^ and significant caregiver burden^8–12^.

Unfortunately, preschool children with recurrent wheezing are not distributed equally across the population. Instead, many of these children are from socially disadvantaged backgrounds^13–21^. For example, wheezing exacerbations are more prevalent in Black or Hispanic preschool children^22–27^ and children experiencing family hardship^28–30^ and systemic structural racism and historic red lining practices^31^. Disadvantaged children may also have increased exposure to pollutants^32,33^ and allergens^34,35^, which can worsen inflammation.

Preschool children with recurrent wheezing are also clinically heterogeneous. While many underlying biological mechanisms likely contribute to differing clinical trajectories^36–39^, the biological features of preschool children with recurrent wheezing have not been well studied. Although features of Type 2 inflammation such as immunoglobulin E (IgE) and eosinophils have been identified as risk factors for the development of asthma in preschool children^40,41^, more than half of this population does not have Type 2 inflammation^42^. Therefore, significant episodes of wheezing continue to occur in many preschool children despite use of therapies directed at Type 2 inflammation such as inhaled corticosteroids^43–45^. The biological features of children with Type 2-low (or non-Type 2) wheezing are not well studied and examinations of other factors aside from IgE and eosinophils that contribute to recurrent wheezing in preschool children are needed.

Untargeted metabolomics identifies the global collection of small molecules generated from metabolic processes. In contrast to targeted metabolomics, which is a hypothesis-driven analysis of a subset of metabolites and utilizes a priori knowledge of metabolic pathways, untargeted metabolomics assesses thousands of metabolites and has emerged as a powerful tool for discovery, hypothesis generation, and identification biological mechanisms^46^. Since there are few studies of metabolomic profiling in preschool children with recurrent wheezing, this study performed untargeted plasma metabolomic profiling on a well-characterized sample of preschool children with recurrent wheezing to identify biological pathways for future investigation. We hypothesized that distinct metabolomic endotypes of preschool children with recurrent wheezing independent of Type 2 inflammation would be identified and that these endotypes would be associated with distinguishing clinical phenotypic features and outcomes.

## Results

**Figure 1:**
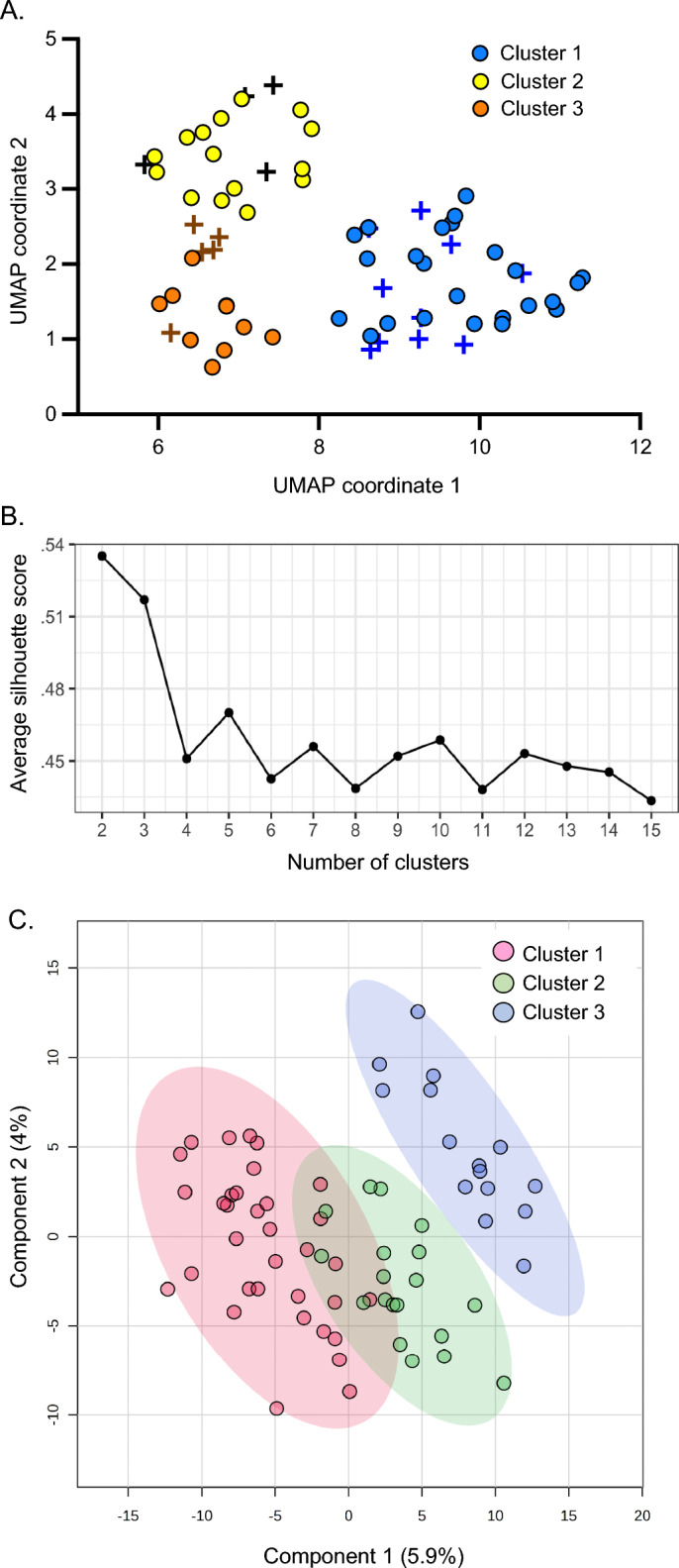
Cluster identification. (**A**) K-means clustering of 1382 plasma metabolites after Uniform Manifold Approximation and Projection (UMAP) for dimension reduction identifies three metabolomic clusters. Individual children are shown as circles (children without aeroallergen sensitization) or as + symbols (children with aeroallergen sensitization). (**B**) Silhouette scores for varying cluster solutions. (**C**) Partial least squares discriminant analysis (PLS-DA) results from all 1382 plasma metabolites, demonstrating the discriminatory ability of metabolite profiles for the cluster groupings.

Untargeted plasma metabolomics results were available from 68 preschool children with recurrent wheezing. The metabolomics dataset included a total of 1382 metabolites, including 1110 compounds of known identity, and 272 compounds of unknown structural identity (unnamed metabolites). Uniform Manifold Approximation and Projection (UMAP)^47^ was performed for dimension reduction using all 1382 metabolites (named and unnamed), yielding two coordinates per patient, and k-means clustering was performed on the UMAP coordinates. After inspection of the elbow plot, a three-cluster solution was identified as the best solution (Fig. 1A). The three-cluster solution, despite having slightly lower yet still acceptable silhouette scores (Fig. 1B), provided more clinically meaningful insights. There was no discernable effect of aeroallergen sensitization status on the clustering results, so children with sensitization were retained in the analysis. Aeroallergen sensitization was associated with higher blood eosinophil counts (*R*^2^ = 0.061, *p* = 0.026) and higher serum IgE concentrations (*R*^2^ = 0.281, *p* < 0.001), but these variables also were not different between the cluster groups (Table 1).

**Table 1:** Demographic and clinical features of the participants. Data represent the median (25th, 75th percentile) or the number of participants (%).

	Cluster 1 Less advantaged, moderately symptomatic N = 34	Cluster 2 Less advantaged, most symptomatic N = 19	Cluster 3 More advantaged, least symptomatic N = 15
Age (months)	34 (27, 54)	29 (21, 37)*	31 (18, 39)
Age of wheezing onset (months)	12 (5, 19)	12 (4, 24)	12 (7, 18)
Males	26 (76.5)	9 (47.4)*	7 (46.7)*
Primary race (self-reported)			
White	16 (47.1)	7 (36.8)	11 (73.3)^#^
Black	17 (50.0)	12 (63.2)	3 (20.0)*^#^
American Indian	1 (2.9)	0	0
Asian	0	0	1 (6.7)
Hispanic ethnicity	5 (14.7)	0	3 (20.0)
Body mass index percentile	65 (44, 87)	79 (65, 93)	39 (22, 63)*^#^
Days since last systemic corticosteroid burst	42 (29, 93)	66 (33, 133)	37 (33, 119)
Highest household education			
Did not complete high school	0	0	1 (6.7)
High school diploma	6 (17.6)	1 (5.3)	7 (10.3)
Some college	11 (32.4)	8 (42.1)	21 (30.9)
Bachelor’s degree	17 (50.0)	10 (52.6)	12 (80.0)*
Wheezing history (past 12 months)			
Number of episodes	5 (3, 7)	5 (4, 6)	3 (2, 5)
Number of corticosteroid bursts	2 (2, 4)	2 (1, 4)	2 (1, 4)
Number of unscheduled visits	3 (2, 4)	3 (2, 4)	2 (1, 4)
Atopic history			
Eczema	13 (38.2)	12 (63.2)	9 (60.0)
Any aeroallergen sensitization	10 (29.4)	4 (21.1)	4 (28.6)
Atopic features			
Total serum IgE (kU/L)	59 (23, 196)	81 (24, 202)	40 (11, 100)
Blood eosinophil count (cells/microliter)	209 (137, 336)	220 (133, 404)	303 (147, 519)
Inhaled corticosteroid use	20 (58.8)	9 (47.4)	6 (40.0)
Current symptoms			
Symptom score (past two weeks)	1.5 (0, 3)	3 (0, 7)*	0 (0, 3)^#^
Symptom score above zero	20 (58.8)	13 (68.4)	5 (33.3)^#^
Wheezing outcomes at 12 months			
Any Emergency Department visit	12 (35.3)	14 (73.7)*	6 (40.0)^#^
Any exacerbation requiring prednisone	28 (82.4)	17 (89.5)	9 (60.0)^#^

*p < 0.05 vs. Cluster 1.

^#^*p* < 0.05 vs. Cluster 2.

Other clinical features of the children in each metabolomics cluster group are shown in Table 1. Metabolomic cluster groups were distinguished by age, sex, race, household educational attainment, and current symptoms. Although body mass index percentiles were slightly higher in children in Cluster 2, obesity defined as a body mass index percentile at or above the 95^th^ percentile was present in only four children in Cluster 1, two children in Cluster 2, and no children in Cluster 3 (*p* = 0.491). Metabolomic cluster groups also differed in measures of social disadvantage obtained from the Childhood Opportunity Index (COI) 2.0^48^, which provides a measure of neighborhood resources and conditions that affect child development (Table 2). The cluster groups also differed in wheezing exacerbation outcomes at 12 months after enrollment (Table 1). The clinical features of the three cluster groups are detailed below.

**Table 2:** Social disadvantage, measured by the residential Childhood Opportunity Index (COI) version 2.0. Data shown are the overall COI score, the three COI domain scores, and significant indicators (*p* < 0.05) within each domain. Data are presented as the median (25^th^, 75^th^ percentile).

Variable	Cluster 1	Cluster 2	Cluster 3
	Less advantaged, moderately symptomatic	Less advantaged, most symptomatic	More advantaged, least symptomatic
	N = 34	N = 19	N = 15
COI overall score^1^	36 (18, 76)	25 (10, 63)	65 (44, 95)*^#^
COI rankings			
Very high	7 (20.6)	3 (15.8)	6 (40.0)
High	3 (8.8)	2 (10.5)	3 (20.0)
Moderate	6 (17.6)	4 (21.1)	4 (26.7)
Low	8 (23.5)	1 (5.3)	1 (6.7)
Very low	10 (29.4)	9 (47.4)	1 (6.7)^#^
COI Education Domain score	56 (16, 83)	27 (7, 67)	76 (55, 98)*^#^
Advanced placement course enrollment (% of 11th and 12th graders)	60 (40, 69)	43 (34, 71)	62 (57, 89)*^#^
Educational attainment (% of adults with a college degree)	25 (17, 45)	30 (18, 42)	50 (32, 65)*^#^
Students eligible for free or reduced lunch (%)	72 (44, 89)	80 (52, 99)	47 (13, 67)*^#^
COI Health and Environment domain score	27 (15, 53)	26 (7, 53)	52 (34, 73)
COI Socioeconomic Domain score	35 (15, 77)	32 (11, 62)	75 (40, 86)*^#^
Households receiving cash assistance or nutrition assistance (%)	14 (4, 26)	17 (5, 27)	5 (2, 14)^#^
Adults with high skill employment (%)	30 (22, 51)	35 (26, 49)	51 (26, 49)*
Median household income (thousands)	51.8 (36.3, 87.1)	46.7 (40.1, 75.6)	89.0 (52.2, 110.1)*^#^
Single-parent households (%)	42 (23, 63)	43 (26, 65)	24 (14, 41)*^#^

^1^Higher COI overall scores and higher COI domain scores reflect better childhood opportunity.

*p < 0.05 vs. Cluster 1; #*p* < 0.05 vs. Cluster 2.

### Features of cluster 1

Thirty-four children were grouped into Cluster 1, termed “less advantaged, moderately symptomatic wheezing.” Children in this cluster were predominantly male (76.5%), with a median age of 34 months and a median wheezing onset at 12 months of age. More than half of the children in this cluster reported a race other than white and 58.8% of the children were currently symptomatic. Children in this cluster overall were socially disadvantaged, with 52.9% of children living in areas classified as having either “low” or “very low” childhood opportunity. At 12 months after enrollment, 70.6% of children in this cluster had an emergency department visit for wheezing and 82.4% received a prednisone burst for a wheezing exacerbation.

### Features of cluster 2

Nineteen children were grouped into Cluster 2, termed “less advantaged, most symptomatic wheezing.” Children in this cluster were 47.4% male and slightly younger, with a median age of 29 months and a median wheezing onset at 12 months of age. Sixty three percent of the children in this cluster reported a race other than white and 68.4% of the children were currently symptomatic. Children in this cluster, like those in Cluster 1, overall were socially disadvantaged, with 52.7% of children living in areas classified as having either “low” or “very low” childhood opportunity. At 12 months after enrollment, 78.9% of children in this cluster had an emergency department visit for wheezing and 89.5% received a prednisone burst for a wheezing exacerbation.

### Features of cluster 3

Fifteen children were grouped into Cluster 3, termed “more advantaged, least symptomatic wheezing.” Children in this cluster were 46.7% male, with a median age of 31 months and a median wheezing onset at 12 months of age. The majority of children in this cluster reported white race and this cluster also had the highest prevalence of household Bachelor’s degree attainment. Compared to Clusters 1 and 2, only 33.3% of the children in this cluster were currently symptomatic. Children in this cluster had least social disadvantage, with only 13.4% of children living in areas classified as having either “low” or “very low” childhood opportunity. Instead, 60% of children in this cluster lived in areas classified as having either “high” or “very high” childhood opportunity. At 12 months after enrollment, 46.7% of children in this cluster had an emergency department visit for wheezing and 60% received a prednisone burst for a wheezing exacerbation.

### Differences in individual metabolites between the clusters

Partial least squares discriminant analysis (PLS-DA), which is a “supervised” version of principal components analysis, was performed on all 1382 plasma metabolites (named and unnamed) to visualize the variability and to assess the discriminatory ability of metabolite profiles for the cluster groupings^49^. With the PLS-DA, component 1 and component 2 explained 5.9% and 4% of the variance, respectively (Fig. 1B). Children in Cluster 1 and Cluster 2 also had more within-group variability than children in Cluster 3, as indicated by the size of the ellipse over the points (Fig. 1C).

**Figure 2:**
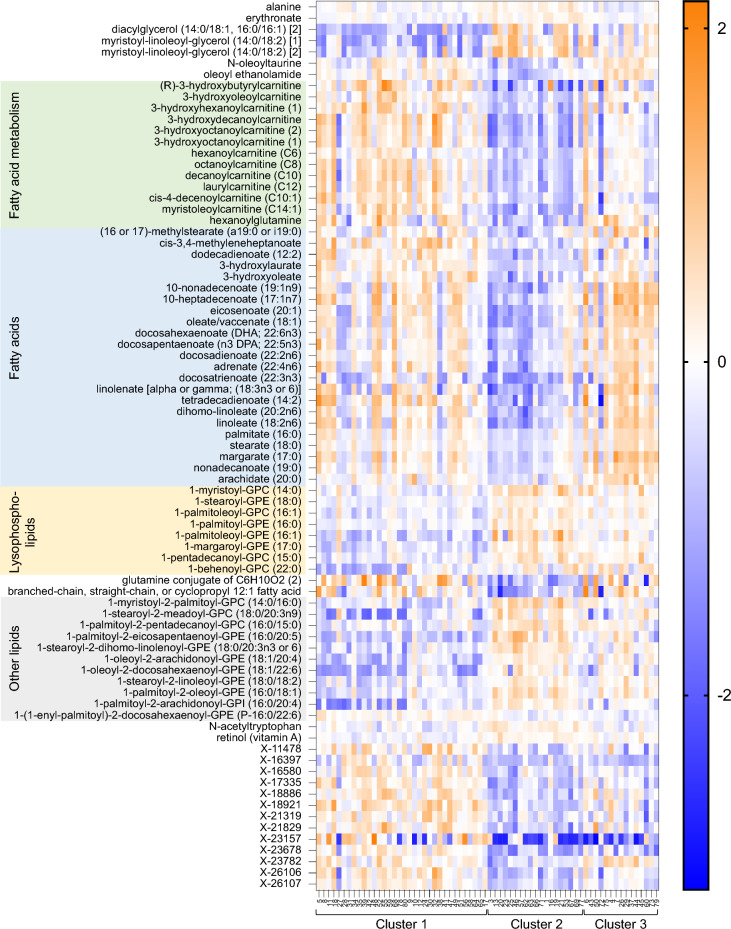
Heatmap of significant metabolites that differ between cluster groups. Normalized concentrations of significant metabolites that differed between the three cluster groups at a Bonferroni-adjusted significance threshold of 0.05 are shown for each cluster group. Higher normalized concentrations are shown in orange and lower normalized concentrations are shown in blue. Metabolites designated with an “X” are unnamed.

**Figure 3:**
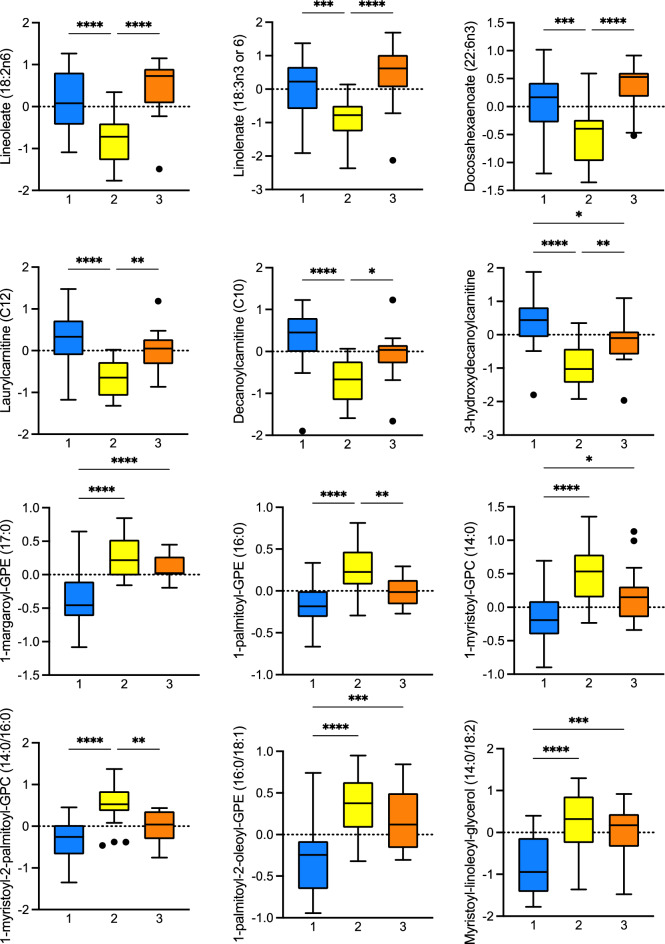
Boxplots of selected normalized lipid metabolites that differ between clusters. Boxplots represent the 25–75th percentile, with horizontal lines at the median. Boxplot whiskers reflect the 5–95th percentile and black circles correspond to outliers. Significant pairwise comparisons between cluster groups are shown with a horizontal line. *p < 0.05, **p < 0.005, ***p < 0.0005, ****p < 0.0001.

To determine the specific plasma metabolites that differed between clusters, analysis of variance was performed using a strict Bonferroni-corrected alpha level of 0.05 to control type 1 error. At a significance threshold of 3.62 × 10^5^, 79 significant metabolites were identified. Thirteen metabolites were unnamed, and 66 metabolites were named. Named metabolites were predominantly lipids and are shown in the heatmap in Fig. 2. Metabolites involved in fatty acid metabolism were lowest in children in Cluster 2 (who were less advantaged and most symptomatic). Fatty acids, including long chain monounsaturated fatty acids, long chain polyunsaturated fatty acids, and long chain saturated fatty acids were also lowest in children in Cluster 2 and highest in children in Cluster 3 (who were more advantaged and the least symptomatic). In contrast, children in Cluster 2 had higher concentrations of lysophospholipids and other lipids including phosphatidylcholines and phosphatidylethanolamines (Fig. 3). Boxplots of selected lipid metabolites are shown in Fig. 3.

### Differential expression of metabolites in each cluster

**Figure 4:**
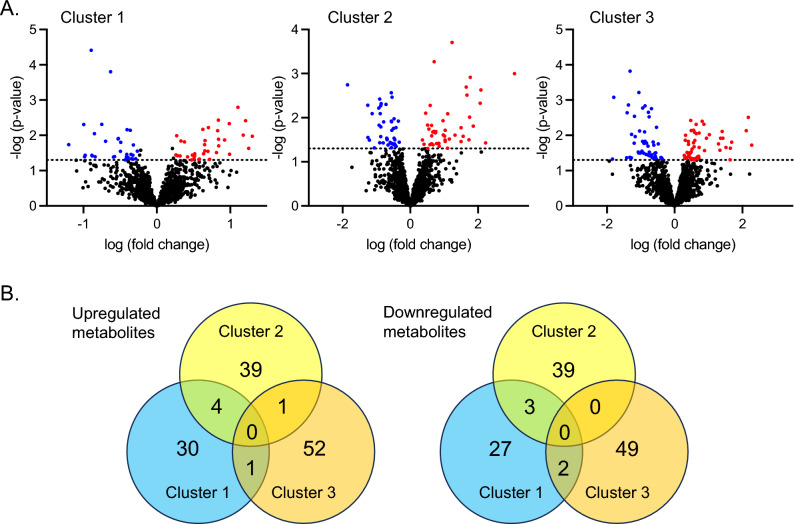
Differential expression of metabolites in each cluster identified by Linear Models for Microarray Data (LIMMA). (**A**) Volcanco plots are shown for each cluster, with upregulated and downregulated metabolites shown in red and blue, respectively. (**B**) Overlap of differentially expressed metabolites between cluster groupings.

Differential expression of metabolites in each cluster was then assessed with the software package Linear Models for Microarray Data (LIMMA), which analyses comparisons between multiple metabolites simultaneously with empirical Bayesian methods, yielding stable results even when the number of participants is small^50^. LIMMA was performed the entire dataset of 1382 metabolites of known and unknown identity using a significance threshold of 0 < 0.05 (without adjustment) and absolute log2 fold change > 0.26. This yielded 67 significant metabolites in Cluster 1 (35 upregulated and 32 downregulated), 86 significant metabolites in Cluster 2 (44 upregulated and 42 downregulated), and 105 significant metabolites in Cluster 3 (54 upregulated and 51 downregulated), with minimal overlap of metabolites between the cluster groups (Fig. 4A,B). Significant metabolites that were upregulated and downregulated in each cluster group are shown in Supplementary Tables S1–S3 and Supplementary Tables S4–S6, respectively.

Similar to the previous analysis, children with Cluster 1, which included children who were less advantaged and moderately symptomatic, had increased expression of fatty acid metabolites and decreased expression of phosphatidylcholines. Additional metabolites identified with LIMMA included upregulated lysine metabolites and downregulated metabolites associated with glycine, serine and threonine metabolism; methionine, cysteine, S-adenosyl methionine and taurine metabolism; tryptophan metabolism; arginine and proline metabolism; and ceramides (Supplementary Table S1, Supplementary Table S4). In Cluster 2, which contained children who were less advantaged and the most symptomatic, LIMMA also identified downregulation of several fatty acid metabolites and upregulation of lysophospholipid and phosphatidylcholine metabolites, similar to the previous analysis. Additional differentially expressed metabolites in Cluster 2 identified by LIMMA included upregulated steroid metabolites and down-regulated metabolites associated with histidine metabolism, sphingomyelins, and sphingosines (Supplementary Table S2, Supplementary Table S5). In Cluster 3, which contained children who were the most advantaged and least symptomatic, LIMMA identified upregulated diacylglycerols and amino acid pathway metabolites and downregulated metabolites associated with fatty acid metabolism, dicarboxylate fatty acids, and secondary bile metabolism (Supplementary Table S3, Supplementary Table S6).

### Metabolic pathway analyses

**Figure 5:**
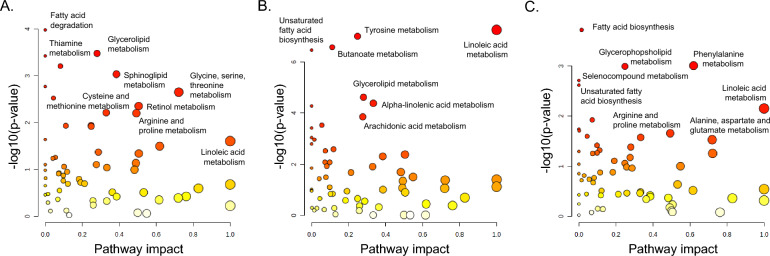
Metabolic pathway analyses. The results of metabolic pathway analyses for (**A**) cluster 1, (**B**) cluster 2, and (**C**) cluster 3. Pathway impact reflects the relative importance of the identified metabolites in the pathway. Larger circles reflect more important pathway impact. Significant pathways at a false discovery rate of 0.05 are shown in red. Non- significant pathways are shown in orange, yellow and white.

Metabolic pathway analyses were then performed for each cluster to understand how the differentially expressed individual metabolites relate to known metabolic pathways. With a false discovery rate of 0.05 as the threshold for significance, 11 pathways were significantly impacted in Cluster 1, which included children who were less advantaged and moderately symptomatic (Supplementary Table S7). The pathway impact, a reflection of the relative importance of the metabolites in the pathway, was highest for glycine, serine and threonine metabolism, followed by retinol metabolism and arginine and proline metabolism (Fig. 5A). In Cluster 2, which contained children who were less advantaged and the most symptomatic, 22 pathways were significantly impacted, and the highest pathway impact was observed for linoleic acid metabolism (Supplementary Table S7, Fig. 5B). In Cluster 3, which contained children who were the most advantaged and the least symptomatic, 5 pathways were significantly impacted, and the highest pathway impact was observed phenylalanine metabolism (Supplementary Table S7, Fig. 5C).

### Exploratory analyses of aeroallergen sensitization and inhaled corticosteroid use

Although aeroallergen sensitization and inhaled corticosteroid use were not different between groups, we performed exploratory analyses with these variables in the entire sample, irrespective of cluster assignment. For this analysis, individual known metabolites were compared with area under the curve (AUC) and log2 fold change. Only three metabolites differed in children with versus without aeroallergen sensitization using a threshold of > 0.75 for AUC and a log2 fold change value > 1.5. These metabolites included choline (AUC = 0.761, log2 fold change = 2.60, *p* = 0.002), acetate (AUC = 0.80, log2 fold change = 1.51, *p* = 2.21E-6), and glycerol (AUC = 0.96, log2 fold change = 1.53, *p* = 1.65E-16) (Supplementary Fig. E1). For the exploratory analysis with inhaled corticosteroid use, no metabolite met these thresholds.

## Discussion

This study performed untargeted plasma metabolomic profiling in a well-characterized sample of preschool children with recurrent wheezing to identify biological pathways for future investigation. With unsupervised k-means clustering of the plasma metabolite data, we identified 3 clusters of preschool children with recurrent wheezing which differed in measures of social disadvantage and respiratory symptoms, but not in features of Type 2 inflammation. Children in Cluster 2 who were less advantaged, the most symptomatic and the most likely to have a wheezing exacerbation by 12 months, had several altered lipids, including lower fatty acids and higher lysophospholipids, phosphatidylcholines and phosphatidylethanolamines. LIMMA also identified differentially expressed steroid metabolites and down-regulated metabolites associated with histidine metabolism, sphingomyelins, and sphingosines in children in Cluster 2. Together, these analyses suggest that that several biological pathways are perturbed in preschool children with recurrent wheezing, which warrant further study.

Although metabolomics studies have been performed in school-age children with asthma^51–55^, very few studies have included preschool children with recurrent wheezing. One study found that preschool children with recurrent and persistent wheezing, compared to children with transient wheezing, had differing urinary metabolomic profiles^56^. Similar to children in our Cluster 2 (who were the most symptomatic), that prior study also found differential expression of phospholipids and fatty acid metabolites in children with persistent versus transient wheezing, suggestive of lipid dysregulation^56^. A separate study of preschool children with recurrent wheezing found that children with Type 2 inflammation (allergic sensitization), compared to children without Type 2 inflammation (no allergic sensitization), had differential expression of several amino acid metabolism pathways in plasma, with lower pyruvate and fumarate and increased glutamine, valine and isobutyric acid^57^. Another study also found pyrimidine metabolism, glycerophospholipid metabolism, and arginine biosynthesis as distinguishing pathways between children who wheezed persistently after respiratory syncytial virus infection compared to those children who did not^58^. However, those previous studies included a small number of participants and utilized different biological samples for the metabolomic analyses.

Our observation of altered fatty acids and lipids in the most symptomatic children in Cluster 2 also has biologic plausibility. Our finding of lower long chain polyunsaturated fatty acids in Cluster 2 supports other literature demonstrating that polyunsaturated fatty acids influence risk of allergic disease. For example, Lee-Sarwar et al. noted that plasma polyunsaturated fatty acid abundances were inversely associated with asthma and recurrent wheezing in children at three years of age^59^. Others have shown that after maternal supplementation with n-3 long chain polyunsaturated fatty acids during pregnancy, the child’s metabolome at age 6 months displays several differences in lipid and amino acid pathways, with a decrease in metabolites in the n-6 long chain polyunsaturated fatty acid and tryptophan pathways and an increase in tyrosine pathways^60^. Children whose mothers received n-3 long chain polyunsaturated fatty acids also had a reduced risk of asthma by age 5^60^. Other lipid pathway alterations (i.e., glycerophospholipids, sphingolipids, and sphingolipid metabolism) have also been reported in asthma and are associated with lung function and airway hyperresponsiveness, while arachidonic acid and linoleic acid metabolism are associated with asthma control^61^.

Our observation of altered metabolomic profiles between less advantaged and more advantaged children is also potentially important. Despite the high prevalence of recurrent wheezing in preschool children, many of these children are from socially disadvantaged backgrounds^13–15^ and this social disadvantage has been shown to influence wheezing outcomes^62^. For example, in the present study, the children in Clusters 1 and 2 with less advantage also likely have different access to food and different exposures, which can influence the metabolome. The comprehensive social impacts on the metabolome (referred to as the “exposome^63^”) have not been well studied but should be considered in future studies of recurrent wheezing in preschool children.

This study does have limitations. First, the statistical and clinical separation of the groups was modest, since thedifferences between the severity of the preschool wheeze are not that prominent between Cluster 1 and Cluster 2. Second, the young age of the children prohibited fasting, so the observed differences in metabolites between the cluster groups may not be solely due to altered catabolism of macromolecules. We cannot exclude other confounding since the clusters differed in several demographic variables including sex and race. The sample size was admittedly small, and this may have prevented detection of other metabolites of importance. Interestingly, some of the metabolites that provided the greatest discrimination between the cluster groups were also unmatched. Therefore, pathway enrichment analyses were not performed on all metabolites because many of these could not be mapped to the existing metabolomics databases. Future studies should place emphasis on less well characterized molecules for potential pathway discovery. We were limited by the cross-sectional analyses and cannot comment on the temporal stability of the metabolome in the cluster groups. Therefore, the specific differentiating metabolites we identified in this study require independent validation. Nonetheless, this work highlights the biologic complexity of recurrent wheezing in preschool children and offers novel metabolites and pathways that may be amenable to future study and intervention.

## Methods

Preschool children 12–59 months of age with recurrent wheezing, defined as a lifetime history of two or more episodes of wheezing, each lasting at least 24 h and requiring repeated treatment with albuterol sulfate, were approached for the study at an outpatient respiratory clinic at Children’s Healthcare of Atlanta, in Atlanta, Georgia. Children were included in the study if they had at least one wheezing episode treated with systemic corticosteroids in the previous 12 months. Children who received systemic corticosteroids within 4 weeks prior to enrollment were rescheduled. Exclusion criteria included premature birth before 35 weeks gestation, immune deficiency, cystic fibrosis, pulmonary aspiration, congenital airway anomalies, and failure to thrive. The Emory University/Children’s Healthcare of Atlanta Institutional Review Board approved the study protocol and informed written consent was obtained for the study from the child’s parent prior to study participation. All study procedures were performed in accordance with the relevant guidelines and regulations in the Declaration of Helsinki.

### Study design and characterization procedures

Preschool children attended a research-only outpatient visit that was rescheduled if the child had an acute illness or a wheezing exacerbation treated with systemic corticosteroids in the preceding two weeks. At the research visit, caregivers completed demographic questionnaires and medical history questionnaires. Respiratory symptom severity was assessed over the previous week with five questions on the severity of cough, wheezing, trouble breathing, activity interference and sleep interference, each of which was scored from zero (none) to five (very severe) and summed, with higher scores reflecting greater respiratory symptoms. Preschool children also submitted blood samples for plasma metabolomics, as well as total serum IgE and blood eosinophil counts, which were performed at a hospital laboratory (Children’s Healthcare of Atlanta, Atlanta, Georgia). At the completion of the visit, the children were followed for 12 months. Outcomes at 12 months included the occurrence of any wheezing episode resulting in an emergency department visit or any wheezing exacerbation requiring treatment with prednisone or equivalent systemic corticosteroids.

### Residential geocoding for childhood opportunity

Residential geocoding was performed as described previously^64^ by mapping participant residential addresses to census tracts using the R package tidygeocoder (R version 4.0.2)^65^. U.S. Census 2020 Geographic Identifiers were mapped to the 2010 GEOIDs for each census tract in Georgia using the R package tigris^66^. Socioeconomic data for each census tract was obtained using the R package tidycensus^67^. The COI 2.0 for 2015^48^, which provides a measure of neighborhood resources and conditions that affect child development, was downloaded and joined to patient data using the census tract GEOID^68^. The COI 2.0 consists of 29 indicators grouped into three domains: education, health and environment, and social and economic resources and opportunities. The COI 2.0 rankings at the level of the state of Georgia were used in the analysis.

### Plasma metabolomics

Blood for metabolomics was collected by venipuncture into an ethylenediaminetetraacetic acid vacutainer tube and centrifuged at 400XG to separate cells from platelet-rich plasma. After initial separation, plasma was centrifuged at 3000XG for 10 min at 4 °C to remove any remaining red blood cells or platelets prior to being frozen at − 80 °C. Plasma metabolomics data acquisition was performed at Metabolon (Morrisville, NC). Sample proteins were precipitated with methanol under vigorous shaking for 2 min (GenoGrinder 2000, Glen Mills, Clifton, NJ) followed by centrifugation. Samples were placed briefly on an evaporator (Zymark TurboVap, Marshall Scientific, Hampton, NH) to remove organic solvent. UPLC-Tandem MS was performed with a liquid chromatograph (Acquity, Waters, Milford, MA) and a high resolution/accurate mass spectrometer interfaced with a heated electrospray ionization source (Q-Exactive, Thermo Scientific, Waltham, MA) and Orbitrap mass analyzer operated at 35,000 mass resolution^69^. The first aliquot was analyzed using acidic positive ion conditions, chromatographically optimized for more hydrophilic compounds (positive early platform). In this method, the extract was gradient eluted from a C18 column (Waters UPLC BEH C18-2.1 × 100 mm, 1.7 µm) using water and methanol, containing 0.05% perfluoropentanoic acid and 0.1% formic acid. The second aliquot was also analyzed using acidic positive ion conditions but was chromatographically optimized for more hydrophobic compounds (positive late platform). In this method, the extract was gradient eluted from the same aforementioned C18 column using methanol, acetonitrile, water, 0.05% perfluoropentanoic acid and 0.01% formic acid and was operated at an overall higher organic content. The third aliquot was analyzed using basic negative ion optimized conditions using a separate dedicated C18 column (negative platform). The basic extracts were gradient eluted from the column using methanol and water, however with 6.5 mM Ammonium Bicarbonate at pH 8. The fourth aliquot was analyzed via negative ionization following elution from a Hydrophilic Interaction Liquid Chromatography column (Waters UPLC BEH Amide 2.1 × 150 mm, 1.7 µm) using a gradient consisting of water and acetonitrile with 10 mM Ammonium Formate, pH 10.8 (polar platform). The MS analysis alternated between MS and data-dependent MS^n^ scans using dynamic exclusion. The scan range varied slightly between methods but covered 70–1000 m/z. Experimental samples were randomized across the platform run with quality control samples spaced evenly among the injections. Peaks were quantified using area-under-the-curve.

### Data analyses

Metabolomics data analyses were performed with R software (version 4.2.3)^70^. Metabolomics data were logarithmically (log2) transformed and then underwent UMAP for dimension reduction^47^, yielding two UMAP coordinates for each participant. K-means clustering was performed based on UMAP coordinates and an elbow plot was used to determine the optimal k number of clusters. The log2-transformed metabolites were then quantile normalized and PLS-DA was performed using MetaboAnalyst 5.0^49^. Significant metabolites that differed between clusters were assessed with general linear models using a Bonferroni-corrected alpha level of 0.05 to control type 1 error. Differential expression of metabolites in each cluster was assessed with the software package LIMMA, which employs a moderated t-statistic, known as parametric Empirical Bayes methodology, for differential expression^50^. Pathway analysis was performed with MetaboAnalyst 5.0^49^ on log-transformed (base 10) and quantile normalized metabolites without data scaling using global test enrichment methods, and relative-betweenness centrality. Pathway analysis integrated pathway enrichment analysis and pathway topology analysis and was restricted to plasma metabolites that could be mapped to the Human Metabolome Database^71^. Other data analyses, including cluster group comparisons of clinical features, were performed with chi-square tests or general liner models with Fisher’s Least Significant Difference post hoc tests, with a significance threshold of 0.05.

### Supplementary Information

Supplementary Information.

## Data Availability

The metabolomics dataset is available from the corresponding author on reasonable request.

## Abbreviations

AUC: Area under the curve
COI: Childhood Opportunity Index
GEOID: Geographic Identifier
IgE: Immunoglobulin E
LIMMA: Linear Models for Microarray Data
MS: Mass Spectroscopy
PLS-DA: Partial least squares discriminant analysis
UMAP: Uniform Manifold Approximation and Projection
UPLC: Ultrahigh Performance Liquid Chromatography
