# Supplementary material for: Plasma metabolomics identifies differing endotypes of recurrent wheezing in preschool children differentiated by symptoms and social disadvantage
Source: Sci Rep. 2024 Jul 9;14:15813. doi: 10.1038/s41598-024-66878-1 (PMC11233605; doi:10.1038/s41598-024-66878-1)
Supplement: Supplementary file 1 — Supplementary Information. [file 41598_2024_66878_MOESM1_ESM.docx]

**Supplementary Information**

**Plasma metabolomics identifies differing endotypes of recurrent wheezing in preschool children differentiated by symptoms and social disadvantage**

Anne M. Fitzpatrick, Ph.D.

Jocelyn R. Grunwell, M.D., Ph.D.

Hina Gaur, B.S.

Seibi Kobara, B.S., M.P.H.

Rishikesan Kamaleswaran, Ph.D.

**Supplementary Table S1.** Differentially expressed, up-regulated metabolites in Cluster 1. Data are organized by pathway. Unnamed metabolites are listed with an “X”.

| Chemical name | Pathway | Sub-pathway | Platform | Log2 fold-change | p-value |
| --- | --- | --- | --- | --- | --- |
| isovalerate (i5:0) | Amino Acid | Leucine, Isoleucine and Valine Metabolism | Neg | 0.506 | 0.041 |
| N2-acetyl,N6-methyllysine | Amino Acid | Lysine Metabolism | Pos Early | 1.208 | 0.004 |
| N6-methyllysine | Amino Acid | Lysine Metabolism | Pos Early | 0.651 | 0.015 |
| 4-acetamidobutanoate | Amino Acid | Polyamine Metabolism | Pos Early | 0.283 | 0.036 |
| indolepropionate | Amino Acid | Tryptophan Metabolism | Neg | 0.813 | 0.028 |
| N-acetylhomocitrulline | Amino Acid | Urea cycle; Arginine and Proline Metabolism | Pos Early | 0.627 | 0.006 |
| androstenediol (3alpha, 17alpha) monosulfate (3) | Lipid | Androgenic Steroids | Neg | 0.580 | 0.045 |
| N-stearoylserine | Lipid | Endocannabinoid | Neg | 0.635 | 0.033 |
| arachidonoylcholine | Lipid | Fatty Acid Metabolism (Acyl Choline) | Pos Late | 0.718 | 0.047 |
| stearamide (18:0) | Lipid | Fatty Acid, Amide | Pos Late | 0.702 | 0.005 |
| 2-hydroxydecanoate | Lipid | Fatty Acid, Monohydroxy | Neg | 0.473 | 0.049 |
| 1-adrenoyl-GPC (22:4) | Lipid | Lysophospholipid | Pos Late | 0.261 | 0.034 |
| 2-stearoyl-GPI (18:0) | Lipid | Lysophospholipid | Neg | 0.515 | 0.038 |
| 1-stearoyl-GPI (18:0) | Lipid | Lysophospholipid | Pos Late | 0.316 | 0.038 |
| 1-palmitoyl-2-(hydroxylinoleoyl)-GPC (16:0/18:2(OH)) | Lipid | Phosphatidylcholine (PC) | Pos Late | 0.485 | 0.036 |
| 1-stearoyl-2-docosapentaenoyl-GPC (18:0/22:5n6)* | Lipid | Phosphatidylcholine (PC) | Pos Late | 0.419 | 0.038 |
| glycerophosphorylcholine (GPC) | Lipid | Phospholipid Metabolism | Pos Early | 0.271 | 0.010 |
| butyrate/isobutyrate (4:0) | Lipid | Short Chain Fatty Acid | Neg | 0.987 | 0.004 |
| valerate (5:0) | Lipid | Short Chain Fatty Acid | Neg | 0.842 | 0.018 |
| propyl 4-hydroxybenzoate sulfate | Xenobiotics | Benzoate Metabolism | Neg | 0.991 | 0.033 |
| 5-hydroxy-2-methylpyridine sulfate | Xenobiotics | Chemical | Neg | 1.301 | 0.010 |
| histidine betaine (hercynine) | Xenobiotics | Food Component/Plant | Pos Early | 0.839 | 0.002 |
| 2-acetamidophenol sulfate | Xenobiotics | Food Component/Plant | Neg | 1.251 | 0.020 |
| levulinoylcarnitine | Xenobiotics | Food Component/Plant | Pos Early | 0.654 | 0.023 |
| X-17351 | Unnamed | Unnamed | Neg | 0.828 | 0.007 |
| X-13658 | Unnamed | Unnamed | Neg | 1.176 | 0.007 |
| X-12283 | Unnamed | Unnamed | Pos Early | 0.840 | 0.011 |
| X-21821 | Unnamed | Unnamed | Neg | 0.922 | 0.013 |
| X-21470 | Unnamed | Unnamed | Neg | 0.656 | 0.014 |
| X-24588 | Unnamed | Unnamed | Neg | 0.329 | 0.014 |
| X-17682 | Unnamed | Unnamed | Neg | 0.377 | 0.015 |
| X-12112 | Unnamed | Unnamed | Pos Early | 0.696 | 0.028 |
| X-24473 | Unnamed | Unnamed | Pos Early | 0.517 | 0.031 |
| X-25937 | Unnamed | Unnamed | Neg | 0.572 | 0.048 |

**Supplementary Table S2.** Differentially expressed, up-regulated metabolites in Cluster 2. Data are organized by pathway. Unnamed metabolites are listed with an “X”.

| Chemical name | Pathway | Sub-pathway | Platform | Log2 fold-change | p-value |
| --- | --- | --- | --- | --- | --- |
| N,N-dimethylalanine | Amino Acid | Alanine and Aspartate Metabolism | Pos Early | 0.742 | 0.022 |
| 3-methoxytyrosine | Amino Acid | Tyrosine Metabolism | Pos Early | 1.649 | 0.002 |
| vanillactate | Amino Acid | Tyrosine Metabolism | Neg | 1.735 | 0.032 |
| 16alpha-hydroxy DHEA 3-sulfate | Lipid | Androgenic Steroids | Neg | 0.705 | 0.001 |
| 5alpha-androstan-3beta,17beta-diol disulfate | Lipid | Androgenic Steroids | Neg | 1.673 | 0.003 |
| andro steroid monosulfate C19H28O6S (1) | Lipid | Androgenic Steroids | Neg | 2.061 | 0.005 |
| androstenediol (3alpha, 17alpha) monosulfate (3) | Lipid | Androgenic Steroids | Neg | 0.586 | 0.005 |
| androstenediol (3beta,17beta) disulfate (1) | Lipid | Androgenic Steroids | Neg | 0.453 | 0.008 |
| androstenediol (3beta,17beta) disulfate (2) | Lipid | Androgenic Steroids | Neg | 1.103 | 0.008 |
| androstenediol (3beta,17beta) monosulfate (1) | Lipid | Androgenic Steroids | Neg | 1.751 | 0.010 |
| androsterone sulfate | Lipid | Androgenic Steroids | Neg | 0.966 | 0.012 |
| dehydroepiandrosterone sulfate (DHEA-S) | Lipid | Androgenic Steroids | Neg | 1.858 | 0.016 |
| epiandrosterone sulfate | Lipid | Androgenic Steroids | Neg | 1.029 | 0.019 |
| cis-3,4-methyleneheptanoylcarnitine | Lipid | Fatty Acid Metabolism (Acyl Carnitine, Medium Chain) | Pos Late | 0.629 | 0.015 |
| 3-hydroxybutyroylglycine | Lipid | Fatty Acid Metabolism (Acyl Glycine) | Pos Early | 1.772 | 0.001 |
| N-acetyl-2-aminooctanoate | Lipid | Fatty Acid, Amino | Neg | 0.667 | 0.021 |
| eicosanedioate (C20-DC) | Lipid | Fatty Acid, Dicarboxylate | Neg | 1.500 | 0.017 |
| eicosenedioate (C20:1-DC) | Lipid | Fatty Acid, Dicarboxylate | Neg | 0.547 | 0.018 |
| 12,13-DiHOME | Lipid | Fatty Acid, Dihydroxy | Neg | 1.234 | 0.000 |
| 21-hydroxypregnenolone disulfate | Lipid | Pregnenolone Steroids | Neg | 3.076 | 0.001 |
| pregnenediol sulfate (C21H34O5S) | Lipid | Pregnenolone Steroids | Neg | 0.975 | 0.025 |
| pregnenetriol disulfate | Lipid | Pregnenolone Steroids | Neg | 1.323 | 0.025 |
| pregnenetriol sulfate | Lipid | Pregnenolone Steroids | Neg | 0.630 | 0.026 |
| pregnenolone sulfate | Lipid | Pregnenolone Steroids | Neg | 1.014 | 0.026 |
| branched-chain, straight-chain, or cyclopropyl 10:1 fatty acid (2) | Partially Characterized Molecules | Partially Characterized Molecules | Neg | 0.511 | 0.015 |
| 4-allylcatechol sulfate | Xenobiotics | Benzoate Metabolism | Neg | 2.086 | 0.002 |
| methyl-4-hydroxybenzoate sulfate | Xenobiotics | Benzoate Metabolism | Neg | 0.752 | 0.021 |
| propyl 4-hydroxybenzoate sulfate | Xenobiotics | Benzoate Metabolism | Neg | 1.562 | 0.026 |
| succinimide | Xenobiotics | Chemical | Neg | 1.150 | 0.031 |
| methyl vanillate sulfate | Xenobiotics | Food Component/Plant | Neg | 0.696 | 0.020 |
| X-12101 | Unnamed | Unnamed | Pos Early | 0.358 | 0.032 |
| X-12822 | Unnamed | Unnamed | Neg | 1.026 | 0.032 |
| X-13695 | Unnamed | Unnamed | Neg | 1.032 | 0.035 |
| X-17346 | Unnamed | Unnamed | Neg | 1.500 | 0.035 |
| X-17653 | Unnamed | Unnamed | Neg | 2.225 | 0.037 |
| X-18899 | Unnamed | Unnamed | Neg | 0.844 | 0.038 |
| X-21470 | Unnamed | Unnamed | Neg | 0.779 | 0.038 |
| X-21815 | Unnamed | Unnamed | Neg | 0.629 | 0.039 |
| X-23583 | Unnamed | Unnamed | Pos Early | 0.406 | 0.040 |
| X-24328 | Unnamed | Unnamed | Neg | 0.581 | 0.041 |
| X-24475 | Unnamed | Unnamed | Pos Early | 0.672 | 0.041 |
| X-24541 | Unnamed | Unnamed | Neg | 0.852 | 0.045 |
| X-24757 | Unnamed | Unnamed | Neg | 1.101 | 0.049 |
| X-25937 | Unnamed | Unnamed | Neg | 0.514 | 0.050 |

**Supplementary Table S3.** Differentially expressed, up-regulated metabolites in Cluster 3. Data are organized by pathway. Unnamed metabolites are listed with an “X”.

| Chemical name | Pathway | Sub-pathway | Platform | Log2 fold-change | p-value |
| --- | --- | --- | --- | --- | --- |
| threonine | Amino Acid | Glycine, Serine and Threonine Metabolism | Pos Early | 0.343 | 0.042 |
| anserine | Amino Acid | Histidine Metabolism | Neg | 2.113 | 0.007 |
| 3-methylhistidine | Amino Acid | Histidine Metabolism | Neg | 1.687 | 0.016 |
| leucine | Amino Acid | Leucine, Isoleucine and Valine Metabolism | Pos Early | 0.303 | 0.045 |
| Tigloylglycine | Amino Acid | Leucine, Isoleucine and Valine Metabolism | Pos Early | 0.617 | 0.046 |
| 5-hydroxylysine | Amino Acid | Lysine Metabolism | Pos Early | 0.722 | 0.022 |
| succinoyltaurine | Amino Acid | Methionine, Cysteine, SAM and Taurine Metabolism | Polar | 0.812 | 0.004 |
| methionine sulfoxide | Amino Acid | Methionine, Cysteine, SAM and Taurine Metabolism | Pos Early | 0.431 | 0.040 |
| N-succinyl-phenylalanine | Amino Acid | Phenylalanine Metabolism | Neg | 0.574 | 0.021 |
| xanthurenate | Amino Acid | Tryptophan Metabolism | Neg | 0.635 | 0.005 |
| Tryptophan | Amino Acid | Tryptophan Metabolism | Pos Early | 0.316 | 0.014 |
| oxindolylalanine | Amino Acid | Tryptophan Metabolism | Pos Early | 0.407 | 0.015 |
| N-acetyltyrosine | Amino Acid | Tyrosine Metabolism | Neg | 0.418 | 0.035 |
| m-tyramine sulfate | Amino Acid | Tyrosine Metabolism | Neg | 0.669 | 0.041 |
| trans-4-hydroxyproline | Amino Acid | Urea cycle; Arginine and Proline Metabolism | Pos Early | 0.423 | 0.032 |
| palmitoyl-oleoyl-glycerol (16:0/18:1) (1) | Lipid | Diacylglycerol | Pos Late | 0.721 | 0.009 |
| oleoyl-oleoyl-glycerol (18:1/18:1) (1) | Lipid | Diacylglycerol | Pos Late | 0.642 | 0.009 |
| palmitoyl-linoleoyl-glycerol (16:0/18:2) (1) | Lipid | Diacylglycerol | Pos Late | 0.597 | 0.014 |
| oleoyl-linoleoyl-glycerol (18:1/18:2) (1) | Lipid | Diacylglycerol | Pos Late | 0.487 | 0.030 |
| oleoyl-arachidonoyl-glycerol (18:1/20:4) (1) | Lipid | Diacylglycerol | Pos Late | 0.532 | 0.046 |
| myristoyl-linoleoyl-glycerol (14:0/18:2) (1) | Lipid | Diacylglycerol | Pos Late | 0.648 | 0.047 |
| linoleoyl-arachidonoyl-glycerol (18:2/20:4) (1) | Lipid | Diacylglycerol | Pos Late | 0.589 | 0.047 |
| palmitoyl-oleoyl-glycerol (16:0/18:1) (2) | Lipid | Diacylglycerol | Pos Late | 0.494 | 0.049 |
| picolinoylglycine | Lipid | Fatty Acid Metabolism (Acyl Glycine) | Neg | 0.515 | 0.025 |
| N-linoleoylglycine | Lipid | Fatty Acid Metabolism (Acyl Glycine) | Neg | 0.714 | 0.043 |
| Propionylglycine | Lipid | Fatty Acid Metabolism (also BCAA Metabolism) | Neg | 1.027 | 0.010 |
| 1-palmitoleoyl-GPC (16:1) | Lipid | Lysophospholipid | Pos Late | 0.475 | 0.004 |
| 1-myristoyl-GPC (14:0) | Lipid | Lysophospholipid | Pos Late | 0.587 | 0.008 |
| 1-margaroyl-GPE (17:0) | Lipid | Lysophospholipid | Pos Late | 0.323 | 0.010 |
| 1-margaroyl-2-oleoyl-GPC (17:0/18:1) | Lipid | Phosphatidylcholine (PC) | Pos Late | 0.285 | 0.037 |
| glycine conjugate of C10H12O2 | Partially Characterized Molecules | Partially Characterized Molecules | Neg | 0.708 | 0.039 |
| 4-hydroxyphenylacetylglutamine | Peptide | Acetylated Peptides | Neg | 0.941 | 0.012 |
| gamma-glutamylisoleucine | Peptide | Gamma-glutamyl Amino Acid | Pos Early | 0.564 | 0.007 |
| gamma-glutamylthreonine | Peptide | Gamma-glutamyl Amino Acid | Pos Early | 0.488 | 0.007 |
| gamma-glutamyltyrosine | Peptide | Gamma-glutamyl Amino Acid | Neg | 0.559 | 0.016 |
| gamma-glutamylmethionine | Peptide | Gamma-glutamyl Amino Acid | Pos Early | 0.409 | 0.018 |
| gamma-glutamylleucine | Peptide | Gamma-glutamyl Amino Acid | Pos Early | 0.462 | 0.032 |
| 2-naphthol sulfate | Xenobiotics | Chemical | Neg | 1.340 | 0.027 |
| N-acetylalliin | Xenobiotics | Food Component/Plant | Neg | 2.174 | 0.003 |
| S-allylcysteine | Xenobiotics | Food Component/Plant | Pos Early | 2.275 | 0.019 |
| 2-oxindole-3-acetate | Xenobiotics | Food Component/Plant | Neg | 0.885 | 0.027 |
| alliin | Xenobiotics | Food Component/Plant | Pos Early | 1.638 | 0.049 |
| X-17676 | Unnamed | Unnamed | Neg | 0.866 | 0.005 |
| X-24757 | Unnamed | Unnamed | Neg | 1.402 | 0.008 |
| X-17690 | Unnamed | Unnamed | Neg | 1.423 | 0.012 |
| X-13658 | Unnamed | Unnamed | Neg | 1.357 | 0.012 |
| X-17328 | Unnamed | Unnamed | Pos Late | 1.294 | 0.018 |
| X-11850 | Unnamed | Unnamed | Neg | 1.517 | 0.022 |
| X-12847 | Unnamed | Unnamed | Neg | 1.645 | 0.023 |
| X-24295 | Unnamed | Unnamed | Neg | 0.369 | 0.031 |
| X-19438 | Unnamed | Unnamed | Neg | 0.406 | 0.040 |
| X-24337 | Unnamed | Unnamed | Neg | 0.464 | 0.044 |
| X-21788 | Unnamed | Unnamed | Neg | 0.345 | 0.046 |
| X-17612 | Unnamed | Unnamed | Neg | 0.591 | 0.046 |

**Supplementary Table S4.** Differentially expressed, down-regulated metabolites in Cluster 1. Data are organized by pathway. Unnamed metabolites are listed with an “X”.

| Chemical name | Pathway | Sub-pathway | Platform | Log2 fold-change | p-value |
| --- | --- | --- | --- | --- | --- |
| cysteine-glutathione disulfide | Amino Acid | Glutathione Metabolism | Pos Early | -0.895 | 0.000 |
| N-acetylthreonine | Amino Acid | Glycine, Serine and Threonine Metabolism | Neg | -0.492 | 0.016 |
| cysteine s-sulfate | Amino Acid | Methionine, Cysteine, SAM and Taurine Metabolism | Polar | -0.496 | 0.028 |
| N-acetyltaurine | Amino Acid | Methionine, Cysteine, SAM and Taurine Metabolism | Neg | -0.883 | 0.038 |
| S-methylcysteine sulfoxide | Amino Acid | Methionine, Cysteine, SAM and Taurine Metabolism | Pos Early | -0.591 | 0.041 |
| S-methylcysteine | Amino Acid | Methionine, Cysteine, SAM and Taurine Metabolism | Neg | -0.392 | 0.042 |
| tryptophan betaine | Amino Acid | Tryptophan Metabolism | Pos Early | -0.985 | 0.040 |
| anthranilate | Amino Acid | Tryptophan Metabolism | Pos Early | -0.345 | 0.046 |
| N-methylproline | Amino Acid | Urea cycle; Arginine and Proline Metabolism | Pos Early | -1.202 | 0.018 |
| N-methylhydroxyproline | Amino Acid | Urea cycle; Arginine and Proline Metabolism | Pos Early | -0.884 | 0.037 |
| beta-cryptoxanthin | Cofactors and Vitamins | Vitamin A Metabolism | Pos Late | -0.576 | 0.040 |
| N-palmitoyl-heptadecasphingosine (d17:1/16:0) | Lipid | Ceramides | Pos Late | -0.321 | 0.019 |
| ceramide (d18:1/17:0, d17:1/18:0) | Lipid | Ceramides | Pos Late | -0.330 | 0.047 |
| glycosyl-N-(2-hydroxynervonoyl)-sphingosine (d18:1/24:1(2OH)) | Lipid | Hexosylceramides (HCER) | Pos Late | -0.630 | 0.000 |
| 1-palmitoyl-2-pentadecanoyl-GPC (16:0/15:0) | Lipid | Phosphatidylcholine (PC) | Pos Late | -0.406 | 0.007 |
| 1-pentadecanoyl-2-linoleoyl-GPC (15:0/18:2) | Lipid | Phosphatidylcholine (PC) | Pos Late | -0.266 | 0.028 |
| 1-myristoyl-2-palmitoyl-GPC (14:0/16:0) | Lipid | Phosphatidylcholine (PC) | Pos Late | -0.353 | 0.035 |
| 1-palmitoleoyl-2-linoleoyl-GPC (16:1/18:2) | Lipid | Phosphatidylcholine (PC) | Pos Late | -0.431 | 0.049 |
| glyco-beta-muricholate | Lipid | Primary Bile Acid Metabolism | Neg | -0.997 | 0.005 |
| glycoursodeoxycholate | Lipid | Secondary Bile Acid Metabolism | Neg | -0.851 | 0.009 |
| deoxycholate | Lipid | Secondary Bile Acid Metabolism | Neg | -0.525 | 0.012 |
| adenosine 5'-diphosphate (ADP) | Nucleotide | Purine Metabolism, Adenine containing | Neg | -0.698 | 0.015 |
| phenylalanylhydroxyproline | Peptide | Dipeptide | Neg | -0.411 | 0.035 |
| valylglutamine | Peptide | Dipeptide | Neg | -0.327 | 0.037 |
| benzoate | Xenobiotics | Benzoate Metabolism | Neg | -0.977 | 0.037 |
| dimethyl sulfone | Xenobiotics | Chemical | Pos Early | -0.414 | 0.041 |
| X-25267 | Unnamed | Unnamed | Pos Early | -0.750 | 0.005 |
| X-11315 | Unnamed | Unnamed | Neg | -0.362 | 0.007 |
| X-24432 | Unnamed | Unnamed | Pos Early | -0.527 | 0.013 |
| X-12027 | Unnamed | Unnamed | Neg | -0.841 | 0.040 |
| X-26054 | Unnamed | Unnamed | Neg | -0.288 | 0.046 |
| X-24306 | Unnamed | Unnamed | Neg | -0.392 | 0.050 |

**Supplementary Table S5.** Differentially expressed, down-regulated metabolites in Cluster 2. Data are organized by pathway. Unnamed metabolites are listed with an “X”.

| Chemical name | Pathway | Sub-pathway | Platform | Log2 fold-change | p-value |
| --- | --- | --- | --- | --- | --- |
| 3-methylhistidine | Amino Acid | Histidine Metabolism | Neg | -1.851 | 0.002 |
| N-acetyl-3-methylhistidine | Amino Acid | Histidine Metabolism | Pos Early | -0.904 | 0.037 |
| 1-methyl-5-imidazolelactate | Amino Acid | Histidine Metabolism | Neg | -1.062 | 0.048 |
| N-acetylproline | Amino Acid | Urea cycle; Arginine and Proline Metabolism | Pos Early | -0.886 | 0.008 |
| nicotinamide | Cofactors and Vitamins | Nicotinate and Nicotinamide Metabolism | Pos Early | -0.562 | 0.003 |
| ceramide (d18:1/17:0, d17:1/18:0) | Lipid | Ceramides | Pos Late | -0.479 | 0.029 |
| N-stearoyl-sphinganine (d18:0/18:0) | Lipid | Dihydroceramides | Pos Late | -0.681 | 0.031 |
| sphingomyelin (d18:0/18:0, d19:0/17:0) | Lipid | Dihydrosphingomyelins | Pos Late | -0.717 | 0.005 |
| behenoyl dihydrosphingomyelin (d18:0/22:0) | Lipid | Dihydrosphingomyelins | Pos Late | -0.534 | 0.038 |
| sphingomyelin (d18:0/20:0, d16:0/22:0) | Lipid | Dihydrosphingomyelins | Pos Late | -0.558 | 0.048 |
| palmitoylcarnitine (C16) | Lipid | Fatty Acid Metabolism (Acyl Carnitine, Long Chain Saturated) | Pos Late | -0.354 | 0.012 |
| margaroylcarnitine (C17) | Lipid | Fatty Acid Metabolism (Acyl Carnitine, Long Chain Saturated) | Pos Late | -0.418 | 0.045 |
| hexanoylcarnitine (C6) | Lipid | Fatty Acid Metabolism (Acyl Carnitine, Medium Chain) | Pos Early | -0.440 | 0.043 |
| palmitoleoylcarnitine (C16:1) | Lipid | Fatty Acid Metabolism (Acyl Carnitine, Monounsaturated) | Pos Late | -0.421 | 0.017 |
| adrenoylcarnitine (C22:4) | Lipid | Fatty Acid Metabolism (Acyl Carnitine, Polyunsaturated) | Pos Late | -0.553 | 0.039 |
| octadecanedioate (C18-DC) | Lipid | Fatty Acid, Dicarboxylate | Neg | -0.559 | 0.012 |
| 10-nonadecenoate (19:1n9) | Lipid | Long Chain Monounsaturated Fatty Acid | Neg | -0.788 | 0.012 |
| palmitoleate (16:1n7) | Lipid | Long Chain Monounsaturated Fatty Acid | Neg | -0.688 | 0.031 |
| arachidate (20:0) | Lipid | Long Chain Saturated Fatty Acid | Neg | -0.553 | 0.012 |
| 1-dihomo-linolenoyl-GPC (20:3n3 or 6) | Lipid | Lysophospholipid | Pos Late | -0.459 | 0.032 |
| 1-palmitoleoyl-GPE (16:1) | Lipid | Lysophospholipid | Pos Late | -0.799 | 0.037 |
| 1-eicosapentaenoyl-GPC (20:5) | Lipid | Lysophospholipid | Pos Late | -0.484 | 0.041 |
| 1-(1-enyl-palmitoyl)-GPE (P-16:0) | Lipid | Lysoplasmalogen | Pos Late | -0.325 | 0.036 |
| (2 or 3)-decenoate (10:1n7 or n8) | Lipid | Medium Chain Fatty Acid | Neg | -0.867 | 0.016 |
| 1-stearoyl-2-meadoyl-GPC (18:0/20:3n9) | Lipid | Phosphatidylcholine (PC) | Pos Late | -0.542 | 0.019 |
| 1-palmitoyl-2-palmitoleoyl-GPC (16:0/16:1) | Lipid | Phosphatidylcholine (PC) | Pos Late | -0.591 | 0.037 |
| 1-stearoyl-2-docosahexaenoyl-GPC (18:0/22:6) | Lipid | Phosphatidylcholine (PC) | Pos Late | -0.467 | 0.039 |
| 1-(1-enyl-palmitoyl)-2-arachidonoyl-GPE (P-16:0/20:4) | Lipid | Plasmalogen | Pos Late | -0.540 | 0.003 |
| 1-(1-enyl-stearoyl)-2-arachidonoyl-GPE (P-18:0/20:4) | Lipid | Plasmalogen | Pos Late | -1.256 | 0.005 |
| deoxycholate | Lipid | Secondary Bile Acid Metabolism | Neg | -0.923 | 0.015 |
| glycolithocholate | Lipid | Secondary Bile Acid Metabolism | Neg | -0.875 | 0.027 |
| sphinganine | Lipid | Sphingolipid Synthesis | Pos Late | -0.495 | 0.018 |
| sphingomyelin (d18:1/25:0, d19:0/24:1, d20:1/23:0, d19:1/24:0) | Lipid | Sphingomyelins | Pos Late | -0.478 | 0.011 |
| sphingosine | Lipid | Sphingosines | Pos Late | -0.903 | 0.004 |
| 5,6-dihydrouracil | Nucleotide | Pyrimidine Metabolism, Uracil containing | Neg | -0.657 | 0.042 |
| perfluorohexanesulfonate (PFHxS) | Xenobiotics | Chemical | Neg | -0.859 | 0.005 |
| perfluorooctanesulfonate (PFOS) | Xenobiotics | Chemical | Polar | -0.929 | 0.006 |
| ectoine | Xenobiotics | Chemical | Pos Early | -1.124 | 0.008 |
| perfluorooctanoate (PFOA) | Xenobiotics | Chemical | Neg | -0.696 | 0.029 |
| 2,3-dihydroxypyridine | Xenobiotics | Food Component/Plant | Pos Early | -1.251 | 0.028 |
| ethyl beta-glucopyranoside | Xenobiotics | Food Component/Plant | Neg | -1.208 | 0.032 |
| X-25267 | Unnamed | Unnamed | Pos Early | -0.873 | 0.005 |

**Supplementary Table S6.** Differentially expressed, down-regulated metabolites in Cluster 3. Data are organized by pathway. Unnamed metabolites are listed with an “X”.

| Chemical name | Pathway | Sub-pathway | Platform | Log2 fold-change | p-value |
| --- | --- | --- | --- | --- | --- |
| cysteine-glutathione disulfide | Amino Acid | Glutathione Metabolism | Pos Early | -0.384 | 0.044 |
| O-acetylhomoserine | Amino Acid | Glycine, Serine and Threonine Metabolism | Polar | -0.887 | 0.008 |
| methylsuccinate | Amino Acid | Leucine, Isoleucine and Valine Metabolism | Polar | -0.352 | 0.049 |
| fructose | Carbohydrate | Fructose, Mannose and Galactose Metabolism | Polar | -1.276 | 0.044 |
| octadecenedioylcarnitine (C18:1-DC) | Lipid | Fatty Acid Metabolism (Acyl Carnitine, Dicarboxylate) | Pos Late | -0.483 | 0.017 |
| 3-hydroxyhexanoylcarnitine (1) | Lipid | Fatty Acid Metabolism (Acyl Carnitine, Hydroxy) | Pos Early | -1.035 | 0.008 |
| (S)-3-hydroxybutyrylcarnitine | Lipid | Fatty Acid Metabolism (Acyl Carnitine, Hydroxy) | Pos Early | -1.195 | 0.017 |
| 3-hydroxybutyroylglycine | Lipid | Fatty Acid Metabolism (Acyl Glycine) | Pos Early | -1.055 | 0.001 |
| trans-2-hexenoylglycine | Lipid | Fatty Acid Metabolism (Acyl Glycine) | Neg | -0.672 | 0.036 |
| 2-butenoylglycine | Lipid | Fatty Acid Metabolism (Acyl Glycine) | Neg | -0.619 | 0.045 |
| sebacate (C10-DC) | Lipid | Fatty Acid, Dicarboxylate | Neg | -0.943 | 0.002 |
| dodecanedioate (C12-DC) | Lipid | Fatty Acid, Dicarboxylate | Neg | -1.162 | 0.011 |
| undecanedioate (C11-DC) | Lipid | Fatty Acid, Dicarboxylate | Neg | -0.633 | 0.015 |
| 2-hydroxysebacate | Lipid | Fatty Acid, Dicarboxylate | Neg | -0.903 | 0.017 |
| azelate (C9-DC) | Lipid | Fatty Acid, Dicarboxylate | Neg | -0.965 | 0.028 |
| hexadecanedioate (C16-DC) | Lipid | Fatty Acid, Dicarboxylate | Neg | -0.583 | 0.032 |
| tetradecanedioate (C14-DC) | Lipid | Fatty Acid, Dicarboxylate | Neg | -0.732 | 0.038 |
| docosadioate (C22-DC) | Lipid | Fatty Acid, Dicarboxylate | Neg | -0.561 | 0.043 |
| 3-carboxy-4-methyl-5-pentyl-2-furanpropionate | Lipid | Fatty Acid, Dicarboxylate | Neg | -0.541 | 0.048 |
| 2S,3R-dihydroxybutyrate | Lipid | Fatty Acid, Dihydroxy | Polar | -0.536 | 0.006 |
| 3-hydroxyhexanoate | Lipid | Fatty Acid, Monohydroxy | Neg | -0.684 | 0.002 |
| 2-hydroxydecanoate | Lipid | Fatty Acid, Monohydroxy | Neg | -0.570 | 0.039 |
| 17alpha-hydroxypregnanolone glucuronide | Lipid | Pregnenolone Steroids | Neg | -1.072 | 0.030 |
| 5alpha-pregnan-diol disulfate | Lipid | Progestin Steroids | Neg | -0.746 | 0.030 |
| glycoursodeoxycholic acid sulfate (1) | Lipid | Secondary Bile Acid Metabolism | Neg | -1.315 | 0.000 |
| tauroursodeoxycholic acid sulfate (1) | Lipid | Secondary Bile Acid Metabolism | Neg | -1.187 | 0.003 |
| glycoursodeoxycholate | Lipid | Secondary Bile Acid Metabolism | Neg | -0.946 | 0.015 |
| isoursodeoxycholate | Lipid | Secondary Bile Acid Metabolism | Neg | -1.321 | 0.042 |
| glutamine conjugate of C6H10O2 (2) | Partially Characterized Molecules | Partially Characterized Molecules | Neg | -1.795 | 0.001 |
| glutamine conjugate of C6H10O2 (1) | Partially Characterized Molecules | Partially Characterized Molecules | Neg | -1.364 | 0.001 |
| glutamine conjugate of C7H12O2 | Partially Characterized Molecules | Partially Characterized Molecules | Neg | -1.414 | 0.043 |
| (S)-a-amino-omega-caprolactam | Xenobiotics | Food Component/Plant | Pos Early | -0.774 | 0.002 |
| methyl glucopyranoside (alpha + beta) | Xenobiotics | Food Component/Plant | Neg | -1.058 | 0.032 |
| X-21736 | Unnamed | Unnamed | Neg | -0.875 | 0.001 |
| X-12101 | Unnamed | Unnamed | Neg | -1.403 | 0.002 |
| X-18922 | Unnamed | Unnamed | Neg | -0.753 | 0.003 |
| X-17674 | Unnamed | Unnamed | Neg | -1.272 | 0.009 |
| X-21319 | Unnamed | Unnamed | Neg | -0.874 | 0.015 |
| X-11478 | Unnamed | Unnamed | Neg | -0.828 | 0.016 |
| X-11847 | Unnamed | Unnamed | Neg | -3.095 | 0.019 |
| X-23276 | Unnamed | Unnamed | Neg | -0.613 | 0.019 |
| X-21829 | Unnamed | Unnamed | Neg | -0.788 | 0.020 |
| X-24947 | Unnamed | Unnamed | Neg | -0.556 | 0.031 |
| X-12839 | Unnamed | Unnamed | Neg | -1.012 | 0.032 |
| X-25936 | Unnamed | Unnamed | Pos Early | -0.910 | 0.032 |
| X-14939 | Unnamed | Unnamed | Neg | -0.841 | 0.035 |
| X-26158 | Unnamed | Unnamed | Neg | -0.786 | 0.036 |
| X-16580 | Unnamed | Unnamed | Pos Early | -0.404 | 0.042 |
| X-18886 | Unnamed | Unnamed | Neg | -0.565 | 0.042 |
| X-24418 | Unnamed | Unnamed | Neg | -0.660 | 0.043 |
| X-11849 | Unnamed | Unnamed | Neg | -1.836 | 0.047 |

**Supplementary Table S7.** Pathway analysis results for each cluster.

| Cluster | Pathway | Total compounds | Hits | p-value | -log10(p) | FDR | Impact |
| --- | --- | --- | --- | --- | --- | --- | --- |
| Cluster 1 | Fatty acid degradation | 39 | 2 | 1.05E-04 | 3.98 | 0.007 | 0.00 |
|  | Glycerolipid metabolism | 16 | 2 | 3.34E-04 | 3.48 | 0.008 | 0.28 |
|  | Thiamine metabolism | 7 | 1 | 3.77E-04 | 3.42 | 7.78E-03 | 0.00 |
|  | Ether lipid metabolism | 20 | 3 | 6.24E-04 | 3.21 | 9.67E-03 | 0.08 |
|  | Sphingolipid metabolism | 32 | 8 | 9.29E-04 | 3.03 | 1.15E-02 | 0.38 |
|  | Neomycin, kanamycin and gentamycin biosynthesis | 2 | 1 | 1.70E-03 | 2.77 | 1.76E-02 | 0.00 |
|  | Glycine, serine and threonine metabolism | 33 | 12 | 2.24E-03 | 2.65 | 1.99E-02 | 0.72 |
|  | Pentose phosphate pathway | 23 | 2 | 3.00E-03 | 2.52 | 2.32E-02 | 0.04 |
|  | Retinol metabolism | 17 | 2 | 4.43E-03 | 2.35 | 3.02E-02 | 0.51 |
|  | Cysteine and methionine metabolism | 33 | 8 | 6.14E-03 | 2.21 | 3.56E-02 | 0.33 |
|  | Arginine and proline metabolism | 36 | 13 | 6.31E-03 | 2.20 | 3.56E-02 | 0.49 |
| Cluster 2 | Linoleic acid metabolism | 5 | 1 | 5.49E-08 | 7.26 | 3.03E-06 | 1.00 |
|  | Tyrosine metabolism | 42 | 9 | 9.76E-08 | 7.01 | 3.03E-06 | 0.25 |
|  | Butanoate metabolism | 15 | 7 | 2.62E-07 | 6.58 | 5.28E-06 | 0.11 |
|  | Biosynthesis of unsaturated fatty acids | 36 | 10 | 3.41E-07 | 6.47 | 5.28E-06 | 0.00 |
|  | Glycerolipid metabolism | 16 | 2 | 2.39E-05 | 4.62 | 2.97E-04 | 0.28 |
|  | Alpha-linoleic acid metabolism | 13 | 2 | 4.12E-05 | 4.38 | 4.26E-04 | 0.33 |
|  | Selenocompound metabolism | 20 | 1 | 5.26E-05 | 4.28 | 4.66E-04 | 0.00 |
|  | Arachidonic acid metabolism | 44 | 1 | 1.38E-04 | 3.86 | 1.07E-03 | 0.28 |
|  | Valine, leucine and isoleucine metabolism | 40 | 9 | 2.96E-04 | 3.53 | 2.04E-03 | 0.06 |
|  | D-amino acid metabolism | 15 | 3 | 3.80E-04 | 3.42 | 2.35E-03 | 0.00 |
|  | Fatty acid degradation | 39 | 2 | 9.07E-04 | 3.04 | 5.11E-03 | 0.00 |
|  | Fatty acid biosynthesis | 47 | 5 | 1.09E-03 | 2.96 | 5.61E-03 | 0.01 |
|  | Nitrogen metabolism | 6 | 2 | 1.60E-03 | 2.80 | 7.63E-03 | 0.00 |
|  | Purine metabolism | 70 | 13 | 2.59E-03 | 2.59 | 1.14E-02 | 0.12 |
|  | Galactose metabolism | 27 | 6 | 2.99E-03 | 2.52 | 1.24E-02 | 0.07 |
|  | Retinol metabolism | 17 | 2 | 4.19E-03 | 2.38 | 1.62E-02 | 0.51 |
|  | Sphingolipid metabolism | 32 | 8 | 4.95E-03 | 2.31 | 1.81E-02 | 0.38 |
|  | Amino sugar and nucleotide sugar metabolism | 42 | 3 | 8.32E-03 | 2.08 | 2.75E-02 | 0.07 |
|  | Fructose and mannose metabolism | 20 | 2 | 8.42E-03 | 2.07 | 2.75E-02 | 0.10 |
|  | Ether lipid metabolism | 20 | 3 | 1.20E-02 | 1.92 | 3.68E-02 | 0.08 |
|  | Cysteine and methionine metabolism | 33 | 8 | 1.25E-02 | 1.90 | 3.68E-02 | 0.33 |
|  | Valine, leucine and isoleucine biosynthesis | 8 | 8 | 1.41E-02 | 1.85 | 3.97E-02 | 0.00 |
| Cluster 3 | Fatty acid biosynthesis | 47 | 5 | 1.91E-04 | 3.72 | 1.18E-02 | 0.01 |
|  | Phenylalanine metabolism | 8 | 5 | 9.85E-04 | 3.01 | 2.11E-02 | 0.62 |
|  | Glycerophospholipid metabolism | 36 | 7 | 1.02E-03 | 2.99 | 2.11E-02 | 0.25 |
|  | Selenocompound metabolism | 20 | 1 | 1.94E-03 | 2.71 | 3.00E-02 | 0.00 |
|  | Biosynthesis of unsaturated fatty acids | 36 | 10 | 2.42E-03 | 2.62 | 3.00E-02 | 0.00 |

**Supplementary Figure E1.** Area under the curve and normalized concentrations of choline, acetate, and glycerol between children without (red) versus with (green) aeroallergen sensitization.
